# Essential Oils and Terpenic Compounds as Potential Hits for Drugs against Amitochondriate Protists

**DOI:** 10.3390/tropicalmed8010037

**Published:** 2023-01-05

**Authors:** Saulo Almeida Menezes, Tiana Tasca

**Affiliations:** Grupo de Pesquisa em Tricomonas, Faculdade de Farmácia e Centro de Biotecnologia, Universidade Federal do Rio Grande do Sul, Av. Bento Gonçalves 9500, Porto Alegre 91501-970, Brazil

**Keywords:** essential oils, terpenes, amitochondriate parasites, chemotherapy, mechanism of action

## Abstract

The human anaerobic or microaerophilic protists *Giardia duodenalis*, *Entamoeba histolytica,* and *Trichomonas vaginalis* are classified as amitochondriate parasites, a group of unicellular organisms that lack canonical mitochondria organelles. These microorganisms suffered adaptations to survive in hostile microenvironments and together represent an increasing threat to public health in developing countries. Nevertheless, the current therapeutic drugs to manage the infections are scarce and often cause several side effects. Furthermore, refractory cases associated with the emergence of parasitic resistance are concerns that guide the search for new pharmacological targets and treatment alternatives. Herein, essential oils and terpenic compounds with activity against amitochondriate parasites with clinical relevance are summarized and insights into possible mechanisms of action are made. This review aims to contribute with future perspectives for research with these natural products as potential alternatives for the acquisition of new molecules for the treatment of amitochondriate protists.

## 1. Introduction

Protozoan parasites include a wide range of eucaryotic organisms, some of which can be important causes of worldwide neglected human diseases. Parasitic infections are a real challenge in developing countries, impacting health and also contributing to morbimortality [1,2,3]. The amitochondriate parasites are a set of unicellular protists that lack mitochondria and often other classical organelles present in eukaryotes. Instead, they possess a remarkable group of reduced structures called mitochondrial-related organelles (MROs), which includes mitosomes, hydrogenosomes, and other mitochondria-like organelles [4,5]. Therefore, whereas mitosomes are present in enteric parasites, *Entamoeba histolytica* and *Giardia duodenalis*, the hydrogenosomes can be found in the urogenital cavity parasite *Trichomonas vaginalis*. This small group of protists inhabit oxygen-deficient microenvironments and have undergone adaptations that do not use the canonical aerobic functions [6].

The drugs of choice for the treatment of these parasitic infections are 5-nitroimidazole derivatives, mainly represented by metronidazole (MTZ). Classically, MTZ functions as a prodrug that needs to be reduced. Parasitic enzymes act in order to reduce the drug in nitro radicals, which disrupt the double-strand DNA and lead to parasite death [7]. However, there are drawbacks to MTZ therapy, such as its adverse side effects: nausea, vomiting, headache, vertigo, diarrhea, disulfiram-like alcohol intolerance, metallic taste in the mouth, and hypersensitivity reactions [8,9]. In recent decades, the failure of therapy has been attributed to the emergence of resistant isolates of *G. duodenalis* and *T. vaginalis* [10,11,12]. For *E. histolytica*, MTZ does not affect the cystic forms in asymptomatic patients and there are no pharmacological options if clinical resistance arises [13]. Thus, the search for new pharmacological targets and therapeutic alternatives is urgent.

Essential oils (EOs) are a group of aromatic, volatile, and lipophilic natural products that are part of the secondary metabolism of vegetal species. In nature, plants produce EOs as a result of physiological stresses [14]. In this way, EOs play a protective role, acting as antimicrobial, insecticide, and allelopathic agents. The chemical composition of EOs is quite diverse, containing mainly terpenes and other non-terpenic compounds [15,16]. The EOs and their related compounds have great commercial value and are widely used in traditional medicine, by phytotherapy practitioners, and in public health services for the treatment of several conditions, including dermatological problems, microbial resistance, and parasitic diseases [17]. Several plant EOs and terpenic compounds (e.g., diterpenes, triterpenes, and other terpenoids) have demonstrated antiparasitic effects [18,19,20,21,22].

In this context, this review summarized the available evidences and mechanisms of the action of EOs and terpenic compounds against the pathogenic amitochondriate parasites *G. duodenalis*, *E. histolytica*, and *T. vaginalis*. The literature cited in the present study was extracted from the US National Library of Medicine (PubMed) combining the terms “*Giardia duodenalis*” or “*Entamoeba histolytica*” or “*Trichomonas vaginalis*” and “essential oil” or “essential oil derivatives” or “terpenes” or “terpenoids” or “diterpenes” or “triterpenes”. We chose all the full-text articles published in the English language evaluating the antiprotozoal activity of essential oils and terpenic compounds using the keywords mentioned above. Publications between 2000 and 2022 were selected to compose the data related to compounds with action against amitochondriate protozoa.

## 2. *Giardia duodenalis*

*Giardia duodenalis* (synonyms *G. lamblia* and *G. intestinalis*) is an extracellular enteric protozoan in the order Diplomonadida that belongs to the Metamonada group [23]. It is one of the main agents responsible for diarrheal diseases worldwide, with more than 300 million cases per year, particularly in low-income and developing countries [24]. The parasite is transmitted mainly through the ingestion of food and water contaminated with fecal origin material, which makes *Giardia* an important pathogen for hospital and food hygiene [25]. Human infections can be asymptomatic or associated with diarrhea, weight loss, distention and abdominal pain, malabsorption, and fatigue. Symptoms are mainly mediated by damage to enterocytes, the loss of the brush borders of intestinal epithelial cells, the loss of microvilli, and the alteration of epithelial barrier function [26].

The biological cycle of *G. duodenalis* is simple and comprises the stages of the cyst, the environmental resistant and highly infective form, and the proliferative trophozoites. The cycle is initiated by the ingestion of cysts present in food or water contaminated with feces. The cysts enter the digestive tract where they are stimulated by the acidic environment of the stomach and by bile and trypsin in the duodenum, differentiating into motile trophozoites [27]. Trophozoites proliferate and adhere to intestinal microvilli using the adhesive disc to prevent peristalsis. Finally, conditions such as increased pH, low cholesterol concentrations, and increased concentration of bile and lactic acid influence the differentiation of trophozoites into infective cysts, which are released in the feces and can be a source of new infections [28].

*Giardia duodenalis* has a simplified subcellular organization, lacking several canonical organelles present in most eukaryotic cells, such as the mitochondria responsible for respiratory metabolism. However, they have mitosomes, MROs that play key roles in the biogenesis and maturation of iron–sulphur (Fe-S) clusters [4]. The energy source for Fe-S clusters’ synthesis remains unclear, but the parasite is equipped with a machinery that includes cysteine desulfurase (IscS), scaffold proteins (IscA2, IscU, and Nfu), the electron transporters ferredoxin and glutaredoxin 5 (Grx5), and the chaperones Hsp-70, Jac-1, and GrpE [29,30]. Furthermore, the therapeutic options available for the treatment of giardiasis include components of the azole group such as MTZ and tinidazole as well as nitazoxanide. Nevertheless, resistance or tolerance to the drugs of choice make therapy for the infection or asymptomatic colonization a real challenge that needs to be overcome [25].

## 3. *Entamoeba histolytica*

*Entamoeba histolytica* is a nonflagellated protozoan in the order Amoebida that infects humans and causes amoebiasis [31]. Amebiasis is a neglected infection and a major public health problem in developing countries, affecting 50 million people per year and causing 100,000 deaths [32]. Its transmission is mediated primarily through the ingestion of fecally contaminated food or water [33]. Most infections are asymptomatic, but the parasite can invade the intestinal wall tissue, manifesting symptoms of mild diarrhea, abdominal pain, dysentery, invasive colitis, and weight loss. In some individuals, extraintestinal symptoms may arise, provoking liver, lung, or brain abscesses, which lead to severe and even fatal risks [34].

This microaerophilic parasite has two stages in its life cycle: the cysts; an infective and dormant form with a high capacity to resist in the hostile external environment; and trophozoites, a non-infectious, motile form capable of invading host tissues [35]. Infection is initiated by the ingestion of food or water contaminated with feces containing cysts. Upon reaching the final portion of the small intestine, trophozoites are generated by the process of excystation and binary fission. Trophozoites colonize the large intestine, where they phagocyte mucous cells and commensal bacteria. Some of these trophozoites undergo morphological and biochemical changes and differentiate into cysts, which are eliminated in feces and may lead to a new infectious process [36].

The species *E. histolytica* is part of the group of anaerobic protozoa that do not possess mitochondria and, together with *Giardia*, contains the remarkably divergent and reduced MRO called mitosome, with enzymes responsible for sulfate activation [6]. These enzymes are essential for cell proliferation and sulfolipids synthesis, such as cholesterylsulfate, a molecule capable of inducing encystation, a crucial process in maintaining the life cycle and disease transmission [37,38,39]. The energy generation in *E. histolytica* occurs in the cytosol and is primarily dependent on substrate-level phosphorylation in glycolysis and fermentation [40]. The treatment of amoebiasis is mainly based on the use of nitroimidazole compounds, such as MTZ, but the growing concern about increasing resistance and tolerance is also one of the disadvantages of this drug [34,41]. Therefore, there is a need to seek new therapeutic targets and alternative strategies for the treatment of amoebiasis.

## 4. *Trichomonas vaginalis*

*Trichomonas vaginalis* is a flagellated protozoan parasite in the order Trichomonadida that affects the human urogenital tract causing trichomoniasis, one of the most common non-viral sexually transmitted infections worldwide [42]. In 2016, the World Health Organization (WHO) estimated the incidence of infection at 156 million new cases per year [43]. However, prevalence data are unknown since it is not a notifiable disease, being subject to underdiagnosis and incorrect diagnosis in clinical practice [44]. 

Currently, about 80% of cases are asymptomatic, both in women and men [45]. The symptoms of trichomoniasis in women include the mild-to-moderate inflammation of the cervix, vagina, and urethra, with odorous vaginal discharge [46]. In men the infection can occur mainly with urethritis or prostatitis [47]. The persistence of parasite contact with epithelial cells leads to the chronicity of the infection and can predispose to cervical and prostate cancer, pelvic inflammatory disease, infertility, preterm delivery, and low birth weight infant [48]. Importantly, *T. vaginalis* infection is also associated with an increased risk of the transmission and acquisition of the human immunodeficiency virus (HIV) [49].

The urogenital pathogen *T. vaginalis* only exists in the infective and motile trophozoite stage and has humans as hosts. The transmission of parasites is mediated mainly through sexual intercourse without the use of condoms [50]. This protist unlike most eukaryotes has an anaerobic metabolism and does not contain mitochondria, harboring hydrogenosomes instead. It is within the hydrogenosomes that the energy metabolism of the parasite occurs with the final production of hydrogen gas [29,51,52]. The treatment of trichomoniasis remains based on the use of 5-nitroimidazole compounds such as MTZ, tinidazole, and secnidazole [53]. However, resistance has become a concern that guides the search for new pharmacological targets and alternative therapies.

## 5. Essential Oils and Terpenic Compounds against Amitochondriate Parasites

**Figure 1:**
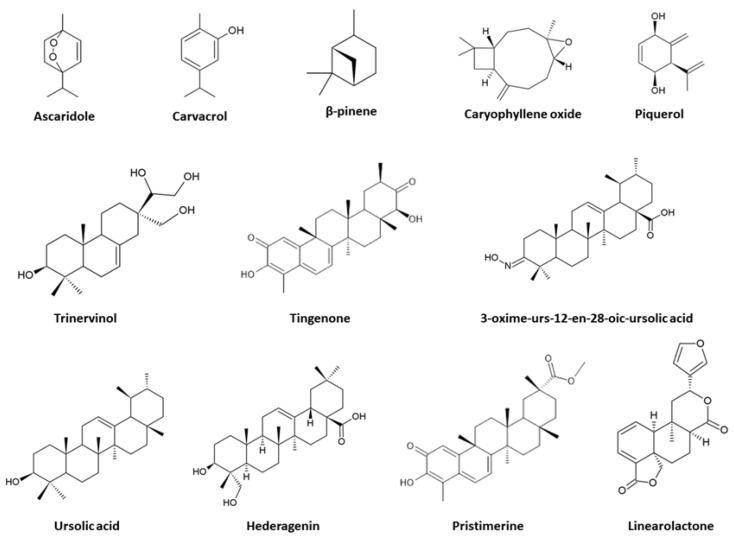
Chemical structures of some promising terpenes and essential oils components against amitochondriate protists.

Several essential oils from plant species and terpenic compounds (diterpenes, triterpenes, and other terpenoids) that show activity against amitochondriate protozoan parasites are presented in this section. The chemical structures of some promising components are shown in Figure 1.

### 5.1. Essential Oils against G. Duodenalis

Essential oils identified to act against *G. duodenalis* are presented in Table 1. The essential oils obtained from *Lavandula angustifolia* and *Lavandula x intermedia* were capable of eliminating all viable trophozoites within 30 min at 10 and 5 mg/mL [54]. The essential oil from the leaves of *Ocimum basilicum* also had an antigiardial effect. The oil (2 mg/mL) was able to kill about 80% of the parasites, in addition to being able to reduce the association index between macrophages and trophozoites [55].

Essential oils rich in phenolic compounds reveal interesting giardicidal activity [56]. The EOs of *Thymbra capitata*, *Origanum virens*, *Thymus zygis* subsp. *sylvestris*, and *Lippia graveolens* (half maximal inhibitory concentration [IC_50_] of 71, 85, 185, and 257 µg/mL, respectively) reduced trophozoite adhesion and were able to kill half of the parasite population in a time-dependent manner. The species *T. capitata* and *O. virens* have carvacrol, a phenolic monoterpene, as the major constituent (74.6% and 68.2%, respectively), while thymol is part of the main constitution of *L. graveolens* and *T. zygis* subsp. *sylvestris* (19.8% and 15.2%, respectively).

**Table 1:** Essential oils from plant species with activity against *Giardia duodenalis*, *Entamoeba histolytica*, and *Trichomonas vaginalis*.

Plant Species/ Botanical Family	Parasitic Organism	Active Concentration	Major Components	Results	References
**Amaranthaceae**					
*Dysphania ambrosioides*	*E. histolytica*	IC_50_: 0.7 mg/mL, 8 and 80 mg/kg	Ascaridole epoxide and cis-Ascaridole	Significant amoebicidal activity	[57]
**Apiaceae**					
*Cuminum cyminum*	*G. duodenalis*	LD_50_: 175 µg/mL	Cuminaldehyde	Significant giardicidal activity	[58]
*Foeniculum vulgare*	*T. vaginalis*	MLC: 1600 μg/mL	E-anethole, fenchone, and ρ-anisaldehyde	Significant trichomonacidal activity	[59]
*Pimpinella anisum*	*G. duodenalis*	LD_50_: 136 µg/mL	trans-Anethole	Significant giardicidal activity	[58]
**Asteraceae**					
*Ageratum conyzoides*	*G. duodenalis*	IC_50_: 35.00 and 89.33 μg/mL (LW–P and FP fractions)	Precocene I, β-caryophyllene, precocene II, α-caryophyllene	Significant giardicidal activity	[60]
*Artemisia absinthium*	*T. vaginalis*	GI_50_: 87.2 μg/mL	cis-epoxycimene, (-)-cis-chrysanthenol, and 3,6-dihydrochamazulene	Significant trichomonacidal activity	[61]
**Fabaceae**					
Brazilian red propolis (Resinous exudates of *Dalbergia ecastophyllum*)	*T. vaginalis*	IC_50_: 100 μg/mL MIC: 500 μg/mL	Methyl eugenol, (E)-β-farnesene, and δ-amorphene	Significant trichomonacidal activity	[62]
**Labiatae**					
*Dracocephalum kotschyi*	*T. vaginalis*	IC_50_: 84.07 μg/mL	Copaene, methyl geranate, geranial, and carvone	Significant trichomonacidal activity; induction of an apoptosis-like cell death on trophozoites	[63]
**Lamiaceae**					
*Lavandula angustifolia*	*T. vaginalis G. duodenalis*	IC_50_: ≤ 1%	Not determined	Significant trichomonacidal and giardicidal activity	[54]
*Lavandula x intermedia*	*T. vaginalis G. duodenalis*	IC_50_: ≤ 1%	Not determined	Significant trichomonacidal and giardicidal activity	[54]
*Ocimum basilicum*	*T. vaginalis* *G. duodenalis*	MLC: 30 µg/mL IC_50_: 2 mg/mL	Linalool, eugenol, and α-Trans-bergamotene	Significant trichomonacidal activity; EO was able to kill almost 80% of *Giardia* trophozoites in 120min	[55,64]
*Origanum virens*	*G. duodenalis*	IC_50_: 85 µg/mL	Carvacrol, γ-Terpinene, and p-Cymene	Significant giardicidal activity	[56]
*Thymbra capitata*	*G. duodenalis*	IC_50_: 71 µg/mL	Carvacrol, p-Cymene, and γ-Terpinene	Significant giardicidal activity	[56]
*Thymus vulgaris*	*E. histolytica*	MIC: 0.7 mg/mL	Not determined	Significant amoebicidal activity	[65]
*Thymus zygis* subsp. *sylvestris*	*G. duodenalis*	IC_50_: 185 µg/mL	p-Cymene, γ-Terpinene, and thymol	Significant giardicidal activity	[56]
**Lauraceae**					
*Cinnamomum verum*	*G. duodenalis*	LD_50_: 108 µg/mL	Cinnamaldehyde	Significant giardicidal activity	[58]
*Laurus nobilis*	*G. duodenalis*	LD_50_: 193 µg/mL	Eucalyptol	Significant giardicidal activity	[58]
*Nectandra megapotamica*	*T. vaginalis*	IC_50_: 98.7 μg/mL	(+)-α-Bisabolol	Significant trichomonacidal activity	[66]
**Myrtaceae**					
*Eucalyptus globulus*	*G. duodenalis*	Antigiardial activity (73.55%) after exposure to 1000 µL/mL	1,8-eucalyptol, α-pinene, α-terpineol acetate	Significant giardicidal activity	[67]
*Eugenia brejoensis*	*T. vaginalis*	MIC: <500 μg/mL	Not determined	Significant trichomonacidal activity	[68]
*Eugenia flavescens*	*T. vaginalis*	MIC: <500 μg/mL	Not determined	Significant trichomonacidal activity	[68]
*Eugenia gracillima*	*T. vaginalis*	MIC: 500 μg/mL IC_50_: 185.6 μg/mL	Not determined	Significant trichomonacidal activity	[68]
*Eugenia pohliana*	*T. vaginalis*	MIC: 500 μg/mL IC_50_: 257.8 μg/mL	Delta-cadinene, bicyclogermacrene, and epi-a-muurolol	Significant trichomonacidal activity against ATCC and fresh clinical isolates; synergistic effect when associated with MTZ	[68]
*Myrciaria floribunda*	*T. vaginalis*	MIC: 500 μg/mL IC_50_: 162.9 μg/mL	Not determined	Significant trichomonacidal activity	[68]
*Psidium myrsinites*	*T. vaginalis*	MIC: 500 μg/mL IC_50_: 179.6 μg/mL	Not determined	Significant trichomonacidal activity against ATCC and fresh clinical isolates; synergistic effect associated with MTZ	[68]
*Syzygium aromaticum*	*G. duodenalis*	LD_50_: 139 µg/mL	Eugenol	Significant giardicidal activity	[58]
**Rutaceae**					
*Atalantia sessiflora*	*T. vaginalis*	IC_50_: 0.016% IC_90:_ 0.03% MLC: 0.06%	Linalool, E-β-caryophyllene, and ledene	Significant trichomonacidal activity	[69]
*Citrus aurantifolia*	*G. duodenalis*	LD_50_: 112 µg/mL	Limonene	Significant giardicidal activity	[58]
**Verbenaceae**					
*Lippia berlandieri*	*G. duodenalis*	LD_50_: 60 µg/mL	Thymol	Significant giardicidal activity	[58]
*Lippia graveolens*	*G. duodenalis*	IC_50_: 257 µg/mL	Thymol, p-Cymene, and caryophyllene oxide	Significant giardicidal activity	[56]
Zingiberaceae					
*Aframomum sceptrum*	*T. vaginalis*	IC_50_: 0.12 µL/mL MLC: 1.72 µL/ml	β-pinene, caryophyllene oxide, and cyperene	Significant trichomonacidal activity	[70]
*Amomum tsao-ko*	*T. vaginalis*	MLC: 44.97µg/mL IC_50_: 22.49 µg/mL	Geraniol (unpublished)	Significant trichomonacidal activity	[19]
*Zingiber officinalis*	*G. duodenalis*	Reduction in cysts (61.15%) at 1000 µL/mL	Geraniol, α-zingiberene, (E,E)-α-farnesene	Significant giardicidal activity	[67]

The EOs from *Eucalyptus globulus* and *Zingiber officinalis* showed antigiardial activity at a concentration of 1000 μL/mL, being able to destroy/inactivate parasite cysts in 73.55 and 61.15%, respectively. Among the major constituents of *E. globulus* oil, 1,8-eucalyptol, α-pinene, and α-terpineol acetate stand out while geraniol, α-zingiberene, and (E,E)-α-farnesene are part of the composition of *Z. officinalis* [67]. Two essential oils obtained from the leaves and flowers of *Ageratum conyzoides* showed a significant effect against the trophozoites of *G. duodenalis* (IC_50_ of 35 and 89.33 μg/mL). The components of these oils included chromenes (precocene I, precocene II, and 6-vinyl-7-methoxy-2,2-dimethylchromene), followed by the traditional sesquiterpenes (β-caryophyllene, α-caryophyllene, and germacrene D) and monoterpenes (α -pinene, camphene, β-pinene, and limonene) [60].

A significant cytotoxic effect against *G. duodenalis* trophozoites was also seen by the essential oils of commercially available species: *Lippia berlandieri* Schauer, *Cinnamomum verum* J. Presl, *Citrus aurantifolia* Swingle, *Pimpinella anisum* Linnaeus, *Syzygium aromaticum* (L.) Merr. and L. M. Perry, *Cuminum cyminum* Linnaeus, and *Laurus nobilis* Linnaeus (IC_50_ of 60, 108, 112, 136, 139, 175, and 193 µg/mL, respectively) [58].

### 5.2. Essential Oils against E. histolytica

There is a huge gap in the literature regarding the biological potential of essential oils against *E. histolytica*. In this sense, the few studies identified are shown in Table 1. The essential oil of air-dried *Thymus vulgaris*, an indigenous plant of the Mediterranean region of Europe known as “garden thyme”, was obtained by hydrodistillation and studied regarding its amoebic activity in comparison to MTZ. The *T. vulgaris* oil presented a minimal inhibitory concentration (MIC) of 0.7 mg/mL and caused growth inhibition of *E. histolytica* trophozoites within 24 h [65]. 

The essential oil of the fresh leaves of *Dysphania ambrosioides* (L.) Mosyakin and Clemants, a herb medicine used as vermifuge, anthelmintic, and emmenagogue, was studied to assess the amoebicidal activity in vitro and compared to MTZ in an amoebic liver abscess hamster model [57]. The oil, rich in ascaridole epoxide (45.5%) and cis-Ascaridole (34.2%), demonstrated an IC_50_ of 0.7 mg/mL with a partial efficacy to produces a consistent protection against the in vivo *E. histolytica* model.

### 5.3. Essential Oils against T. vaginalis

Essential oils identified to act against *T. vaginalis* are presented in Table 1. The *Lavandula angustifolia* and *Lavandula x intermedia* EOs were capable of eliminating all viable trophozoites at concentrations of 10 and 5 mg/mL [54]. The essential oil of *Aframomum sceptrum* K. Schum was reported to exert a remarkable trichomonicidal activity with a minimal lethal concentration (MLC) of 1.72 µL/mL and an IC_50_ of 0.12 µL/mL [70]. The main components of *A. sceptrum* essential oil were β-pinene, caryophyllene oxide, and cyperene.

The essential oil obtained by the vapor pressure of *Artemisia absinthium* L. showed in vitro trichomonacidal activity and also a trypanocidal effect [61]. The aromatic and medicinal plant *A. absinthium* is of ethnopharmacological interest, and the main compounds of essential oil found in this study were cis-epoxycimene, (-)-cis-chrysanthenol, and 3,6-dihydrochamazulene. The essential oil from *Ocimum basilicum* Linn (L.) showed in vitro activity against the trophozoites of *T. vaginalis* with an MLC of 30 µg/mL, comparable to the MTZ positive control at 40 µg/mL [64]. Transmission electron microscopy (TEM) results showed considerable damage to the membrane system around parasites [64]. The Chinese spice and traditional medicine component *Amomum tsao-ko* Crevost et Lemaire also reported in vitro trichomonicidal effects against two different *T. vaginalis* isolates. The values of the MLC and IC_50_ of *A. tsao-ko* were 44.97 mg/mL and 22.49 mg/mL for the Tv1 isolate and 89.93 mg/mL and 44.97 mg/mL for the Tv2 isolate. Although the activity was lower than that of MTZ, it was still more potent than Jieeryin, a well-known therapeutic agent in Chinese medicine for the treatment of vaginitis caused by *T. vaginalis* [19].

In recent decades, Brazilian red propolis (BRP) obtained from resinous exudates of *Dalbergia ecastophyllum* has been studied for chemical characterization and to obtain new bioactive compounds. Therefore, BRP essential oil was evaluated against *T. vaginalis* and demonstrated to be able to inhibit the growth of parasites with an IC_50_ of 100 μg/mL and MLC of 500 μg/mL. The kinetic growth curve showed a 36% decrease in trophozoites growth after 12 h of exposure to 500 μg/mL, with the induction of complete parasite death after 24 h [62]. The essential oil of *Foeniculum vulgare* presented a trichomonacidal activity with an MLC of 1600 μg/mL. The determined composition of *F. vulgare* oil revealed E-anethole (88.41%) as the main compound, followed by fenchone and ρ-anisaldehyde [59].

The anti-*T. vaginalis* activity from the essential oil of *Nectandra megapotamica* was demonstrated and the oil rich in (+)-α-bisabolol (93.7%) had an IC_50_ of 98.7 μg/mL and an MIC of 500 μg/mL. However, in safety assays to verify compatibility with mammalian cells, the essential oil showed cytotoxicity towards Vero cell lineage and human erythrocytes [66]. The essential oil of *Atalantia sessiflora* Guillauminin fresh leaves was studied to assess the antimicrobial and trichomonicidal activities [69]. The oil was rich in linalool, E-β-caryophyllene, ledene, α-humulene, and L-α-terpineol. The results of the IC_50_, IC_90_, and MLC values were 0.016, 0.03, and 0.06% (*v*/*v*), respectively, after 48 h of incubation.

Recently, the anti-*T. vaginalis* potential of essential oils obtained from Myrtaceae species from the Brazilian Caatinga was investigated. Particularly, *Psidium myrsinites* and *Eugenia pohliana* oils showed an MIC of 500 μg/mL and IC_50_ of 179.6 and 257.8 μg/mL, respectively. Both EOs showed synergism when associated with MTZ and the assay against the vaginal epithelial cell line showed moderate cytotoxicity [68]. Finally, the essential oil of *Dracocephalum kotschyi* presented an IC_50_ of 84.07 μg/mL against *T. vaginalis*, without showing cytotoxicity on the J774.A1 cell line. Interestingly, flow cytometry assays showed that trophozoites exposed to 100 and 700 μg/mL concentrations of the oil developed an apoptosis-like cell death [63].

### 5.4. Terpenic Compounds against G. duodenalis

Terpenic compounds with anti-*G. duodenalis* effects are shown in Table 2. The most active compounds identified were the triterpenoids pristimerine and tingenone, with IC_50_ values of 0.11 and 0.74 µM, respectively. However, both are cytotoxic, which limits their use [71]. In another study, a clerodane diterpene, linearolactone (LL), isolated from *Salvia gesneriflora*, presented an IC_50_ of 28.2 μM against *G. duodenalis* trophozoites [72]. A significant giardicidal effect was also seen to be exerted by ent-Kaurene glycosides (41.9–48.9 μM) [73], thymol derivatives (IC_50_ = 151.1–167.4 μM) [18,74], trinervinol (IC_50_ = 2.03 μg/mL), and piquerol (IC_50_ = 2.42 μg/mL) [75]. Other promising anti-*G. duodenalis* essential oil derivatives are eugenol, linalool [55], dihydroartemisinin [76], 7-hydroxy-3,4-dihydrocadalene, 7-hydroxycalamenene [77], incomptine A [78], anethole, carvacrol, cinnamaldehyde, cuminaldehyde, eucalyptol, limonene, and thymol [58].

Furthermore, the terpene retinol was found to improve host defenses against *Giardia* infections in a clinical trial conducted with Brazilian children. The prospective randomized, double-blind, placebo-controlled trial study involved 79 school children treated with retinol (100,000 IU for children <12 months and 200,000 IU for children at least 12 months old; N = 39) or placebo (tocopherol 40 IU; N = 40). At the end of the study, the prevalence of new infections, especially with *Giardia*, was significantly decreased with retinol intervention [89].

### 5.5. Terpenic Compounds against E. histolytica

The few studies that evaluated the anti-*E. histolytica* effects of terpenic compounds are summarized in Table 2. The most active compounds identified were the terpene-based 1,4,2-dioxazoles: 2-(3-(benzo[d][1,3]dioxol-5-yl)-5-methyl-1,4,2-dioxazol-5-yl)pyridine and 2-(3-(benzo[d] [1,3]dioxol-5-yl)-1,4,2-dioxazol-5-yl) pyridine, with IC_50_ values of 1.00 and 1.03 µM, respectively, alongside other semisynthetic compounds. Toxicological studies of the active compounds in H9c2 rat cardiac myoblasts did not show toxicity [90]. The clerodane diterpene LL, presented an IC_50_ of 22.9 μM against *E. histolytica* trophozoites [72]. The LL diterpene was able to induce an apoptosis-like effect with the intracellular production of reactive oxygen species (ROS). Moreover, the compound altered the actin cytoskeleton and reduced the development of amoebic liver abscesses (ALA) in the in vivo experiments [87].

A significant amoebicidal effect was also showed by the *Ageratina cylindrica* diterpenoids, ent-15β-(β-L-fucosyloxy)kaur-16-en-19-oic acid β-D-glucopyranosyl ester and ent-15β-(4-Acetoxy-β-L-fucosyloxy)kaur-16-en-19-oic acid β-D-glucopyranosyl ester (IC_50_ = 43.3 and 49.5 μM) [73] and thymol derivatives (IC_50_ = 184.9 μM) [74]. Other promising anti-*E. histolytica* essential oil derivatives are ascaridole [57], piperitone [88], incomptine A [78], and the pentacyclic triterpenoid isoarborinol [85].

### 5.6. Terpenic Compounds against T. vaginalis

Terpenic compounds with anti-*T. vaginalis* activity are shown in Table 2. The anti-*T. vaginalis* properties of geraniol, a major component of the essential oil of *A. tsao-ko* Crevost et Lemaire, which belongs to the Zingiberaceae family, was investigated [19]. Geraniol is a monoterpene widely used as a component of fragrances and has many biological potentials, including as an antimicrobial, antioxidant, antitumoral, and insecticidal agent. Geraniol showed in vitro anti-*T. vaginalis* activity against two different isolates; however, it was 4-8-fold less active than the essential oil of *A. tsao-ko*, which could be attributed to the interaction and synergism of the compounds in the essential oil mixture [19]. The two sesquiterpenes, caryophyllene oxide and β-pinene, components of *A. sceptrum* K. Schum oil (Zingiberaceae), also showed marked in vitro anti-*T. vaginalis* properties, with MLC/IC_50_ values of 0.625/0.16 mg/mL and 1.25/0.44 mg/mL, respectively [70].

The aerial parts of *Azorella yareta* Hauman were used to obtain the diterpenoids named 13β-hydroxyazorellane, mulinolic acid, mulin-11,13-dien-20-oic acid, azorellanol, and 13α-hydroxyazorellane [79]. All tested compounds showed in vitro anti-*T. vaginalis* activity with LC_50_ values ranging 40–120 µM, which is, however, almost 10 times higher than that of MTZ (LC_50_ = 6.6 µM). The pentacyclic triterpenoid hederagenin isolated through a bioguided fractionation of the dichloromethane extract of the traditional African medicinal plant *Cussonia holstii* demonstrated trichomonicidal activity with an IC_50_ of 2.8 μM [83]. 

Structural modifications in betulinic acid, a pentacyclic triterpenoid isolated from branks of *Palatanus acerifolia*, resulted in the production of two derivatives capable of reducing the viability of *T. vaginalis* trophozoites by 100% with an MIC of 50 μM. However, the significant cytotoxicity of these derivatives was observed against the HeLa, HMVII, and Vero cell lines [20]. A piperazine derivative from betulinic acid was also shown to be an efficient trichomonicidal molecule with an MIC of 91.2 μM, and a kinetic growth curve performed with treated parasites showed the complete inhibition of trophozoites in 2 h of incubation [80].

It is noteworthy to consider the search for triterpene derivatives as a promising approach for the development of new trichomonicidal agents. In this sense, the anti-*T. vaginalis* activity of ursolic acid was performed and the compound showed an MIC of 50 μM against the MTZ-sensitive isolate and, even more, showed an MIC of 12.5 μM against a resistant isolate [91]. The semisynthetic ursolic acid derivative, 3-oxime-urs-12-en-28-oic-ursolic acid, was tested against *T. vaginalis* and presented MIC values of 25 μM and 12.5 μM against a sensitive and resistant isolate, respectively. When this compound was associated with MTZ, a strong synergistic effect could be noted. In addition, it presented very low cytotoxicity against the Vero cell line, a normal cell line, reducing only about 15% of the viability [81].

The medicinal and aromatic plant *Foeniculum vulgare* (Apiaceae) had its botanical extracts and essential oil evaluated against *T. vaginalis*. The oil presented the peculiar phenylpropanoid trans-anethole as major component and it was evaluated for the anti-*T. vaginalis* activity against five parasite isolates. The in vitro susceptibility assay showed that the compound had an MLC of 1600 μg/mL, which was much higher than that of the drug of choice, MTZ [59].

## 6. Mechanisms of Action of Essential Oils and Terpenic Compounds against Amitochondriate Parasites

There is a wide range of studies evaluating the antiparasitic activity of natural products. However, when we funnel to essential oils and terpenic compounds the amount of information is scarce. Furthermore, the mechanism of action of these products is a subject of discussion. The amitochondriate parasites have a unique cell biology due to the lack of mitochondria and other canonical organelles. Instead, mitosomes and hydrogenosomes play a key role in the biogenesis of iron–sulfur clusters, sulfate activation, ATP, and hydrogen gas production [4,6,92]. Altogether, these organelles represent important and attractive targets for the acquisition of new drugs aiming at the improvement of chemotherapy against these parasites, since they have different metabolisms from the mitochondria and are not found in mammalian cells.

Compounds that act directly on mitosomes/hydrogenosomes could be highly selective to parasites without causing harm to host cells [93]. It is important to consider that these organelles are essential to parasite survival in hostile microenvironments and therefore contribute to the establishment of infection. However, these structures have not been targeted in natural products research and should be further explored.

Undoubtedly, the most striking feature of essential oils and terpenic compounds is their hydrophobicity. The lipophilic nature of these compounds allows them to cross cell membranes [94]. Thus, they have the ability to interact with intercellular components, impairing their functions and leading to cell death due to the increased permeation of cytoplasmic components [95]. It is well stated that terpenes can disrupt the lipid composition of the bacterial cell wall, leading to cellular membrane disintegration, protein denaturation, and the leakage of cytoplasmic material, which produce cell lysis and cell death. In addition, they can also interfere with oxidative phosphorylation or oxygen uptake in bacteria, thereby inhibiting bacterial growth [96].

**Figure 2:**
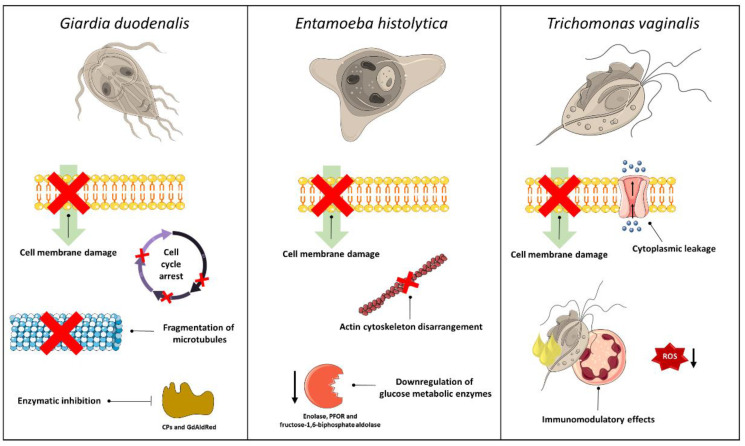
Possible mechanisms of action displayed by essential oils and terpenic compounds on amitochondriate parasites.

The mechanisms of action attributed to the compounds presented in this review can be summarized in three main aspects: (i) cell membrane damage, (ii) the modulation of the immune response, and (3) enzymatic inhibition (Figure 2). It is important to note that the mechanisms by which EOs act have not yet been studied in detail and there are a large number of chemical compounds present in these mixtures. Therefore, when discussing EOs, it is likely that the pharmacological action is attributed not to a single component, but to the synergism between compounds.

Cell membrane damage: Ultrastructural analyses using TEM and scanning electron microscopy (SEM) are the most common protocols used to verify the direct effect of natural products and isolated molecules on parasites’ cell membranes, cytoskeleton, and general morphology. The exposure of the *T. vaginalis* clinical isolate Tv2 to *A. tsao-ko* essential oil and its major component, geraniol, resulted in drastic changes in the protist morphology. The appearance of vacuoles, disappearance of ribosomes, dilation of the endoplasmic reticulum, and disintegration of other organelles, in addition to partial damage to the plasma membrane and the leakage of cytoplasmic content, were observed [19]. Considerable damage to the membrane system and extensive vacuolization of the cytoplasm of a *T. vaginalis* clinical isolate was also observed after exposure to *O. basilicum* essential oil [64].

Trophozoites of *T. vaginalis* ATCC 30236 isolate exposed to the terpenoid ursolic acid demonstrated morphological and ultrastructural alteration [91]. The SEM showed untreated parasites with regular shape, but after 2 h of treatment critical effects on the parasite membrane were observed. The classic piriform shape was replaced by a rounded format, with the appearance of membrane projections and holes, indicating that the parasite entered in cell death [91]. Considering that cytoadherence is an essential step for *T. vaginalis* to establish infection, further parallel adhesion assays should be conducted in order to clarify whether EOs or terpenic compounds could impact on the attachment capacity of this parasite.

Furthermore, the use of TEM and flow cytometry techniques revealed that dihydroartemisinin (DHA) is capable of inducing changes in the morphology and cell cycle of *G. duodenalis* clinical isolate C2 [76]. Exposure to DHA caused damages to the cytoskeleton, with the fragmentation of microtubules in the ventral discs originating lamellar structures. Additionally, the parasite cell cycle was stopped in several phases (GO/G1, S and G2+M), resulting in the suppression of cell growth and differentiation [76]. The exposure to *Syzygium aromaticum* essential oil and eugenol led to changes in the shape of the trophozoites of the *G. duodenalis* WB strain, with the emergence of autophagic vesicles, internalization of flagella and ventral disc, membrane bubbles, and intracellular and nuclear clearing. In addition, eugenol was able to inhibit the adhesion of trophozoites from the third hour of incubation [82]. Importantly, the attachment of *G. duodenalis* to intestinal cells is essential for the colonization of the small intestine and is considered a prerequisite for parasite-induced enterocyte dysfunction and clinical disease [4,97]. This process is mediated by a specific structure in *Giardia*, the ventral disc. Further assays are needed to investigate if essential oils or terpenic compounds are able to disrupt this organelle. Thus, the inhibition of adherence induced by *S. aromaticum* essential oil and eugenol are important to control infection in the treatment of giardiasis.

The neo-clerodane diterpenoid LL isolated from *Salvia polystachya* presents several antiparasitic activities. The effects of this compound on the *G. duodenalis* WB strain ultrastructure were analyzed by TEM. The LL treatment induced the appearance of perinuclear and periplasmic spaces, a decrease in peripheral vesicles, and an accumulation of glycogen granules in cytoplasm. The flow cytometry analysis showed a concentration-dependent increase in cells at necrosis, suggesting that LL could be inducing a necrotic-like cell death in *G. duodenalis* trophozoites [86]. The treatment of *E. histolytica* HM1-IMSS strain trophozoites with LL induced ultrastructural alterations, with a loss of vacuolar structures and a decrease in the trophozoite size. Together with the increase in ROS production, annexin V binding, and DNA fragmentation assays, a possible mechanism of apoptosis-like cell death is suggested. The LL treatment was also able to disarrange the actin cytoskeleton of *E. histolytica*, which was corroborated by a docking analysis showing that it binds to the allosteric site of actin [87]. This finding should be further explored in the next steps of research, since actin cytoskeleton signaling pathways are essential for *E. histolytica* host tissue invasion and therefore represent an attractive target against amebiasis.

Modulation of the immune response: The establishment and success of infection for parasites to complete their life cycle involves several molecular and biochemical mechanisms so they can evade the immune response. In this way, parasites are able to overcome innate immunity and resist elimination mediated by adaptive immunity [98,99]. Unfortunately, the modulation of the immune response is an approach that still needs to be explored in studies with natural products against parasites. In this scenario, drugs capable of stimulating the immune response may be a promising approach in conjunction with antiparasitic agents. Neutrophils stimulated by a *T. vaginalis* ATCC 30236 isolate treated with the betulinic acid derivative N-{3-[4-(3-Aminopropyl)piperazinopropyl]terbutylcarbamate}-3-O-hexanoylbetulinamide and MTZ showed a decreased production of ROS. This finding indicates that this compound plays an immunomodulatory role in the ROS production by neutrophils, causing an anti-inflammatory effect and possibly damaging the cell signaling process, helping in the process of the death of trophozoites [20].

Enzymatic inhibition: The availability of protozoan parasites’ entire genomes along with robust data from omics tools enables the discovery of enzymatic inhibitors. Compounds that target protein structures are essential in interrupting parasite survival. An assay investigated the enzymatic profile of peptidases in the trophozoite lysates of the *G. duodenalis* Portland-I strain, treated or not with *O. basilicum* essential oil and linalool using gelatin-SDS-PAGE [55]. A total proteolytic inhibition of peptidases with molecular masses ranging from 29.6 to 64 kDa in treated parasites was observed. Further characterization with E-64 confirmed that the compounds were able to inhibit cysteine peptidases (CPs). The crucial role of peptidases in the biology of parasitic protozoa is well established since they are enzymes that catalyze the hydrolysis of peptide bonds by the cleavage of amide linkages in a macromolecular or oligomeric protein. In particular, CPs present a cysteine-thiol group at the active site of the enzyme and are widely distributed in pathogenic protists participating in the processes of cell invasion, nutrient acquisition, evasion of the immune response, surface protein processing, induction of the inflammatory responses, and parasitic egress [100,101]. This suggests that CPs from amitochondriate protozoan parasites could be targets for EOs and terpenic compounds.

The treatment of *E. histolytica* HM1-IMSS strain trophozoites with the sesquiterpene lactone incomptine A evidenced a profile of 21 proteins differentially expressed using a proteomic approach based on two-dimensional gel electrophoresis and mass spectrometry (ESI-MS/MS) [84]. Among these proteins, the glycolytic enzymes enolase, pyruvate:ferredoxin oxidoreductase (PFOR), and fructose-1,6-biphosphate aldolase were down-regulated. This finding suggests that incomptine A could be inducing a disruption in trophozoites’ glucose metabolism. Additionally, these results were reinforced by TEM ultrastructural observations, with the increasing accumulation of cytoplasmic glycogen granules [84]. The trophozoites of *E. histolytica* do not possess Krebs cycle and oxidative phosphorylation enzymes, so the energy generation is exclusively dependent on substrate-level phosphorylation in glycolysis and fermentation [40]. Further experiments are required in order to find differences between parasite and host glycolytic enzymes that aid the design of specific inhibitors of the *E. histolytica* glycolytic pathway.

The aldose reductase (AldRed) is an NADPH-dependent cytosolic oxidoreductase enzyme that catalyzes the reduction of glucose to sorbitol in the polyol pathway of glucose metabolism. Using a molecular docking approach, it was investigated whether the diterpene LL was able to interact and inhibit *G. duodenalis* AldRed (GdAldRed, PDB ID: 3KRB) after observations of glycogen granules accumulation in LL-treated trophozoites. The use of “blind” and “active site-direct” tools showed that GdAldRed is a viable target for LL, possible by dimer dissociation and active-site inhibition. This suggests that giardicidal effects of LL could be in part by the affinity with this glycolytic enzyme [86].

## 7. Conclusions

This review compiled a range of essential oils and terpenic compounds that can act against amitochondriate protozoan parasites. Collectively, these microorganisms represent a real threat to areas with intense poverty, the marginal communities of urban centers, and rural areas in developing countries. Current data reveal that essential oils and related compounds could serve as promising agents for prospecting new antiparasitic molecules. One limitation faced during the data analysis was the lack of uniformization in the units (IC_50_, IC_90_, MIC, MLC) and methods used to evaluate parasites’ viability. This gap impairs the comparison among studies and attempts to equalize data may lead to misunderstood conclusions. This point is really a challenge in the field and requires the optimization/validation of parasite viability assays with consequent unit uniformization. In addition, the vast majority of the studies were performed in vitro and in vivo tests are needed in order to confirm the therapeutic potential of these compounds against parasitic infections. In addition, safety and toxicity assays should be performed along with the elucidation of the mechanisms of action and identification of specific targets, in order to provide insights that could be useful for biotechnological applications. It is expected that this review could stimulate future studies on the chemical diversity and bioactivity of essential oils aiming to control *Giardia duodenalis*, *Entamoeba histolytica*, and *Trichomonas vaginalis* infections.

## Figures and Tables

**Table 2:** Essential oil derivatives with activity against *Giardia duodenalis*, *Entamoeba histolytica*, and *Trichomonas vaginalis*. (To be continued).

Terpene/Terpenoids	Protist	Active Concentration	Effect/ Mechanism of Action (MoA)	References
(8S)-8,9-epoxy-6-hydroxy-l0-benzoyloxy-7-oxothymol isobutyrate	*G. duodenalis* *E. histolytica*	IC_50_: 167.4 μM IC_50_: 184.9 μM	Growth inhibition/ unknown MoA	[74]
[2-(2-[(2-methylpropanoyl)oxy]-4-{[(2-methylpropanoyl)oxy]methyl}phenyl)oxiran-2-yl]methyl benzoate	*G. duodenalis*	IC_50_: 164.5 μM	Antiprotozoal properties associated with the presence of 2 methylpropanoate groups at C(7) and/or C(3)	[18]
[2-(5-hydroxy2-[(2-methylpropanoyl)oxy]-4-{[(2-methylpropanoyl)oxy]methyl}phenyl)oxiran-2-yl]methyl benzoate	*G. duodenalis*	IC_50_: 151.1 μM	Antiprotozoal properties associated with the presence of 2 methylpropanoate groups at C(7) and/or C(3)	[18]
13b-hydroxyazorellane	*T. vaginalis*	LD_50_: 100 μM	Growth inhibition/ unknown MoA	[79]
13α-hydroxyazorellane	*T. vaginalis*	LD_50_: 119 μM	Growth inhibition/ unknown MoA	[79]
Piperazine derivative from betulinic acid	*T. vaginalis*	MIC: 91.2 μM	Growth inhibition/ unknown MoA	[80]
7-hydroxy-3,4-dihydrocadalene	*G. duodenalis*	IC_50_: 15.3 μg/mL IC_90_: 23.69 μg/mL	Growth inhibition/morphological and ultrastructural changes (dense material accumulation around nuclei, vacuolization of the cytoplasm, lateral membrane disruption, and ventral disc fragmentation)	[77]
7-hydroxycalamenene	*G. duodenalis*	IC_50_: 13.5 μg/mL IC_90_: 24.21 μg/mL	Growth inhibition/ unknown MoA	[77]
(E)-3-oximeurs-12-en-28-oic acid	*T. vaginalis* (MTZ sensitive and resistant isolate)	MIC: 25 µM (MTZ-sensitive) and 12.5 µM (MTZ-resistant)	Synergic effect with MTZ against the resistant isolate; growth inhibition/unknown MoA	[81]
Anethole	*G. duodenalis*	LD_50_: 134.99 µg/mL	Growth inhibition/ unknown MoA	[58]
Ascaridole	*E. histolytica*	80mg of Ascaridole decreased significantly the number of trophozoites	Growth inhibition/ unknown MoA	[57]
Azorellanol	*T. vaginalis*	LD_50_: 40.5 µM	Growth inhibition/ unknown MoA	[79]
N-{3-[4-(3-Aminopropyl)piperazinopropyl]terbutylcarbamate}-3-O-hexanoylbetulinamide	*T. vaginalis*	MIC: 50 µM	ROS production by neutrophils was reduced and showed anti-inflammatory effect	[20]
Betulinic acid derivative	*T. vaginalis*	MIC: 50 µM	ROS production by neutrophils was reduced	[20]
Carvacrol	*G. duodenalis*	LD_50_: 31.92 µg/mL	Growth inhibition/ unknown MoA	[58]
Caryophyllene oxide	*T. vaginalis*	IC_50_: 0.16 mg/mL; MLC: 0.625 mg/ml	Growth inhibition/ unknown MoA	[70]
Cinnamaldehyde	*G. duodenalis*	LD_50_: 76.42 µg/mL	Growth inhibition/ unknown MoA	[58]
Cuminaldehyde	*G. duodenalis*	LD_50_: 141.16 µg/mL	Growth inhibition/ unknown MoA	[58]
Dihydroartemisinin	*G. duodenalis*	LD_50_: 200 μg/mL	Morphological and ultrastructural changes; damages to the cytoskeleton-impaired parasites to complete cell division at different stages, resulting in suppression of growth and differentiation	[76]
ent-15β-(β-L-Fucosyloxy)kaur-16-en-19-oic acid β-D-glucopyranosyl ester	*G. duodenalis E. histolytica*	IC_50_: 41.9 μM IC_50_: 43.3 μM	Growth inhibition/ unknown MoA	[73]
ent-15β-(4-Acetoxy-β-L-fucosyloxy)kaur-16-en-19-oic acid β-Dglucopyranosyl ester	*E. histolytica*	IC_50_: 49.5 μM	Growth inhibition/ unknown MoA	[73]
ent-15β-(3-Acetoxy-β-L-fucosyloxy)kaur-16-en-19-oic acid β-Dglucopyranosyl ester	*G. duodenalis*	IC_50_: 48.9 μM	Growth inhibition/ unknown MoA	[73]
Eucalyptol	*G. duodenalis*	LD_50_: 265.43 µg/mL	Growth inhibition/ unknown MoA	[58]
Eugenol	*G. duodenalis*	LD_50_: 104.04 µg/mL IC_50_: 101 µg/mL	Morphological and ultrastructural changes (membrane blebs, precipitates in the cytoplasm, fragmentation of ventral disc, autophagic vacuoles, and swelling of peripheral vacuoles)	[55,58,82]
Geraniol	*T. vaginalis*	MLC: 342.96 µg/mL IC_50_: 171.48 µg/ml	Morphological and ultrastructural changes (autophagic vacuoles formation, organelles disintegration, partial cell membrane damaging, and cytoplasmic leakage)	[19]
Hederagenin	*T. vaginalis*	IC_50_: 2.8 μM	Growth inhibition/ unknown MoA	[83]
Incomptine A	*G. lamblia* *E. histolytica*	IC_50_: 11.8 μg/mL IC_50_: 2.6 μg/mL	The proteomic profile evidenced a down-regulation of enolase, pyruvate:ferredoxin oxidoreductase (PFOR), and fructose-1,6-biphosphate aldolase; ultrastructural alterations (increase in cytoplasmic glycogen granules)	[78,84]
Isoarborinol	*E. histolytica*	85.2% of growth inhibition at 0.3 mg/ml	Growth inhibition/ unknown MoA	[85]
Limonene	*G. duodenalis*	LD_50_: 127.59 µg/mL	Growth inhibition/ unknown MoA	[58]
Linalool	*G. duodenalis*	MIC: 300 μg/ml	Cysteine proteases inhibition	[55]
Linearolactone	*G. duodenalis E. histolytica*	IC_50_: 28.2 μM IC_50:_ 22.9 μM	Induction of a necrotic-like death with ultrastructural alterations and the prediction of GdAldRed as a likely target in *G. duodenalis* trophozoites; induction of an apoptosis-like death with the intracellular production of ROS and alteration of the actin cytoskeleton in *E. histolytica* trophozoites; reduction in the development of amoebic liver abscesses (ALA) in vivo	[72,86,87]
Piperitone	*E. histolytica*	IC_50_: 25 µg/mL	Growth inhibition/ unknown MoA	[88]
Piquerol	*G. duodenalis*	IC_50_: 2.42 μg/mL IC_90_: 8.74 μg/mL	Growth inhibition/ unknown MoA	[75]
Pristimerine	*G. duodenalis*	IC_50_: 0.11 μM	Growth inhibition/ unknown MoA	[71]
Retinol/vitamin A	*Giardia* spp.	Retinol at 100,000–200,000 IU	*Giardia* spp. infections were significantly reduced in the group treated with retinol when compared with the placebo group, suggesting an improvement of the host defenses against *Giardia* infections.	[89]
2-(3-(benzo[d][1,3]dioxol-5-yl)-5-methyl-1,4,2-dioxazol-5-yl) pyridine	*E. histolytica*	IC_50_: 1.00 μM	Growth inhibition/ unknown MoA	[90]
2-(3-(benzo[d][1,3]dioxol-5-yl)-1,4,2-dioxazol-5-yl) pyridine	*E. histolytica*	IC_50_: 1.03 μM	Growth inhibition/ unknown MoA	[90]
3-(benzo[d][1,3]dioxol-5-yl)-5-(furan-2-yl)-5-methyl-1,4,2-dioxazole	*E. histolytica*	IC_50_: 1.10 μM	Growth inhibition/ unknown MoA	[90]
3-(benzo[d][1,3]dioxol-5-yl)-5-(furan-2-yl)-1,4,2-dioxazole	*E. histolytica*	IC_50_: 1.09 μM	Growth inhibition/ unknown MoA	[90]
2-(5-(benzo[d][1,3] dioxol-5-yl)-1,4,2-dioxazol-3-yl)pyridine	*E. histolytica*	IC_50_: 1.06 μM	Growth inhibition/ unknown MoA	[90]
5-(benzo[d][1,3]dioxol-5-yl)-3-(furan-2-yl)-1,4,2-dioxazole	*E. histolytica*	IC_50_: 1.05 μM	Growth inhibition/ unknown MoA	[90]
Thymol	*G. duodenalis*	LD_50_: 21.44 µg/mL	Growth inhibition/ unknown MoA	[58]
Tingenone	*G. duodenalis*	IC_50_: 0.74 μM	Growth inhibition/ unknown MoA	[71]
Trans-anethole	*T. vaginalis*	MLC: 1600 μg/mL	Growth inhibition/ unknown MoA	[59]
Trinervinol	*G. duodenalis*	IC_50_: 2.03 μg/mL IC_90_: 13.03 μg/mL	Growth inhibition/ unknown MoA	[75]
Ursolic acid	*T. vaginalis*	MIC: 50 μM IC_50_: 35.3 μM	Morphological and ultrastructural changes (disruption of shape with membrane projections and holes, and undulating membrane and flagella was displayed)	[91]
β-pinene	*T. vaginalis*	IC_50_: 0.44 mg/mL; MLC: 1.25 mg/ml	Growth inhibition/ unknown MoA	[70]

## Data Availability

Not applicable.
